# The internet user profile of Italian families of patients with rare diseases: a web survey

**DOI:** 10.1186/1750-1172-8-76

**Published:** 2013-05-16

**Authors:** Alberto E Tozzi, Rita Mingarelli, Eleonora Agricola, Michaela Gonfiantini, Elisabetta Pandolfi, Emanuela Carloni, Francesco Gesualdo, Bruno Dallapiccola

**Affiliations:** 1 Epidemiology Unit, Bambino Gesù Children’s Hospital, IRCCS, Piazza S. Onofrio 4, 00165 Rome, Italy

**Keywords:** Internet, Telemedicine, Rare diseases

## Abstract

**Background:**

The use of the Internet for searching and sharing health information and for health care interactions may have a great potential for families of children affected with rare diseases. We conducted an online survey among Italian families of patients with rare diseases with the objective to describe their Internet user profile, and to explore how Internet use affects their health decisions.

**Methods:**

All members of UNIAMIO FIMR, a federation of associations of patients with rare diseases, were invited via mail to participate in an online questionnaire including questions on socio-demographic and clinical information, Internet use with a specific focus on health, and impact of web information on health behaviors. Logistic regression models were used to explore the effect of socio-demographic variables and Internet user profile on dependent variables representing the impact of web information on health behaviors. Multiple imputation by chained equations was applied.

**Results:**

A total of 516 parents of patients with rare diseases completed the online questionnaire. Mean age was 43 years. 87% of respondents accessed the Internet daily, 40% through their smartphones. 99% had an email account, 71% had a Facebook account. 66% participate in an online forum on health. 99% searched for information on disease characteristics, 93% on therapy, 89% on diagnosis, 63% on alternative therapies, 62% on nutrition and 54% on future pregnancies. 82% stated that web information increased comprehension of the disease, 65% that it improved management of the disease. For 52% web information increased his or her anxiety. 62% recognized diagnosis, 69% discussed online information with their physician. People participating in forums more frequently stated that Internet information was useful for recognizing their child’s disease (OR 1.68; 95%CI 1.08-2.63) and for improving its management (OR 1.77; 95%CI 1.11-2.81).

**Conclusion:**

Italian parents of patients with rare diseases are active Internet users, engaged in information search and in online communities.

Physicians, health care facilities and health agencies have a great opportunity to engage in online interactions for empowering families of patients of children affected with rare diseases.

## Background

The high penetration of the Internet in the general population of industrialized countries promises to favor more frequent interactions between patients and the health care system, with the potential to improve the quality of care and to increase patients’ participation in clinical decisions [1]. The use of the Internet for health care interactions may represent a necessity in patients with rare diseases to better manage their complex health needs [2]. Patients and caregivers also show an increasing attitude and will to actively participate in health care processes [1]. Patients may provide self-tracking measures of vital signs and other biological or behavioral parameters that can be transmitted through the Internet and may become a rich information basis for clinical decisions [3]. On the other hand, patients and caregivers may actively search for information relevant to their disease which is not easily available and may be unknown to their physicians [4,5]. Furthermore, the creation of online communities of patients and caregivers who share information on their disease may enhance their empowerment and may facilitate participation of patients in clinical trials [6-8].

A notable initiative aimed to engage patients with rare diseases in communities is RareConnect, created by EURORDIS and NORD, a platform where individuals and families affected by rare diseases can connect with each other and find helpful resources [9].

Health care systems may not yet be ready to embrace patient participation models, and health professionals may even not be aware of the potential for interactive communication with patients and caregivers that already exists, or may fear an active role of their patients or their caregivers in clinical decisions [2].

Knowledge of patient’s behavior on the web is crucial to set up appropriate health communications strategies. First, knowing how individuals affected by a certain disease search for health information on the Internet may help providing better information through the most used web channels. Secondly, knowing how patients and their families interact through the Internet and understanding how these behaviors impact on their health decisions may be important for programming their active engagement in the health care process [10].

The Internet penetration in the general Italian population is 58.7% as of mid 2012 [11] and Italy posted the highest growth in Internet users in Europe between April 2011 and April 2012, at 24% [12]. Use of the Internet is more frequent in families with children (79%) [13]. A recent survey reports that 32% of the general population uses the Internet for searching for health information; of these, 90% searches for specific diseases, and 14% uses chat, forum, and web communities to discuss health topics [14].

Most Italian associations of patients with rare diseases joined together in a federation, UNIAMO Federazione Italiana Malattie Rare (FIMR), which is part of the European Organization for Rare Diseases (EURORDIS), and which currently counts more than 100 associations, and nearly 20000 subscribers with more than 600 rare diseases represented [15].

We conducted a survey among Italian families of patients with rare diseases in partnership with UNIAMO FIMR with the objective to describe their Internet user profile, and to explore how Internet use affects their health decisions.

## Methods

An invitation to participate in an online survey on Internet behavior was sent via e-mail by all UNIAMO FIMR federated associations to their members.

The survey was conducted with a standardized questionnaire published online on a dedicated website from July to October 2012. During this period, monthly reminders were sent to federated associations that solicited their members to participate in the survey. The questionnaire was specifically directed to parents of patients affected with a rare disease, and included questions on socio-demographic information, general information on patients’ disability caused by the disease, and information on Internet use with a specific focus on health perceptions and behaviors. An additional part of the questionnaire focused on the impact of web information on health behaviors. The last section of the questionnaire was dedicated to free comments. The entire survey was conducted anonymously on the web and participants were not requested any information on their disease. The study was approved by the ethical committee of the Bambino Gesù Children’s Hospital.

We described absolute frequencies and percentages of socio-demographic variables, and of those regarding the use of the Internet, including its impact on health behaviors. We then explored through logistic regression models the effect of socio-demographic variables and Internet user profile on different variables representing the impact of information found on the Internet on health perceptions and behaviors. In particular, we built 6 different models, in which the dependent variables were as follows: 1) information found on the Internet increased knowledge of my child’s disease; 2) improved management of my child’s disease; 3) increased my anxiety; 4) made me discuss such information with physician; 5) helped in suspecting the diagnosis; 6) made me change my child’s food habits. Among the independent variables we considered: if the respondent was a mother or a father; child’s age; parent's age and education; if the child with a rare disease had either a physical or an intellectual disability; if the respondent had an account in a social network; if the respondent run a blog; if the respondent participated in forums on his or her child’s disease; if the respondent communicated electronically with the physician (via email, Facebook or telemedicine); if the respondent searched the web for information on nutrition, alternative therapies, and planning of future pregnancies.

We first performed the analysis excluding missing data. Since a further analysis revealed that lacking information was missing at random, we applied multiple imputation by chained equations to improve the efficiency of the analysis [16].

Adjusted odds ratios and their 95% confidence intervals were used as measures of effect. Stata software was used for data analysis.

## Results

### Population included in the study

An estimated number of 2300 emails were sent to the UNIAMO members. A total of 516 (22%) parents of patients with rare diseases completed the online questionnaire. The description of their socio-demographic characteristics is reported in Table 1.

**Table 1:** Sociodemographic characteristics of participants in the survey

	**N**	**%**	**Mean**	**SD**	**Min**	**Max**
Mothers	347	67.6				
Parent age			42.7	9	22	79
High school degree or higher	449	87				
Employed	385	74.9				
Child age			10.3	9	0	47
Motor disability	259	49.2				
Intellectual disability	175	33.3				
Both motor and intellectual disability	118	22.4				

SD = standard deviation.

The mean age of responders was 43 years with a range from 22 to 79 and the majority of them were mothers. One third had a university degree and 75% were employed. While most fathers had a job (93%), 66% of mothers were employed. Of note, 31% of parents declared they had to leave their job because of their child’s disease.

The mean age of children with rare diseases was 10 years and they ranged from neonates to adults (max age 47 years). Nearly 70% of patients born to respondents had a disability.

### Internet access and use

Table 2 shows the profile of respondents regarding Internet access and use. The vast majority of respondents had an Internet access at home and 74% had access to the Internet at their workplace. A total of 87% of respondents accesses the Internet daily. More than 65% of respondents had a webcam. More than 40% of responders used their cellular phone for connecting to the Internet and more than 40% had used an app for smartphones. Nearly 27% used a tablet.

**Table 2:** Internet access and use by age group

	**Age group**								
	**<40 yrs (N = 211)**		**40-54 yrs (N = 264)**		**>55 yrs (N = 42)**		**Total (N = 571)**		**p**
	N	%	N	%	N	%	N	%	
Connects to the Internet at home	195	95.1	240	97.2	37	94.8	472	96.1	0.487
Connects to the Internet at workplace	99	71.2	152	76.8	14	63.6	265	73.8	0.279
Has a webcam	132	65.0	155	63.8	30	76	317	65	0.275
Connects to the Internet through a cell phone	91	44.8	104	42.6	8	20.5	203	41.8	0.017
Connects to the Internet through a tablet	50	25.3	70	29.3	7	18	127	26.7	0.307
Has used an app for smartphones	94	47.5	116	44.6	2	8	212	44.1	0.002
Has an email account	199	98.5	245	100	36	94.7	480	99	0.008
Has a Facebook account	163	77.3	177	67	26	61.9	366	70.8	0.022
Has a Twitter account	42	19	44	16.7	3	7.1	89	17.2	0.128
Has a Skype account	104	49.3	125	47.3	24	57.1	253	48.9	0.494
Runs a blog	15	7.5	17	6.9	3	7.9	35	7.3	0.962
Participates in online forum on health	131	67	161	66.3	21	60.0	313	66.2	0.710

Almost all respondents owned an email address, more than 70% had a Facebook account, and 49% had a Skype account, while 17% had a Twitter account. Seven percent of interviewed parents run a blog and 66% participated in online forums on health. Among those who participated in health forums, 81% shared clinical information on their child’s disease within the online community.

Selecting responders with children >6 years of age, parents reported that 61% of their children used the Internet.

Regarding exchange of information and communication with their physician, half respondents reported to communicate with their physician through email (50%), 9.0% through Facebook, and 2% through other social networks. On the other hand, telemedicine was used infrequently (2%).

### Information search on the internet and impact on health decisions

Almost all respondents used the Internet for searching information on their child’s disease as illustrated in Table 3. A total of 93% of responders searched for information on treatments, nearly 90% on diagnosis, and nearly 90% on specialty physicians or hospitals. Parents also frequently searched for information on prevention of complications, nutrition, physical activities, and immunizations. Furthermore, 68% searched for a second opinion, 63% searched for alternative therapies, and 54% searched for information on future pregnancies. Finally, nearly 3% of responders stated they bought medicines online.

**Table 3:** Information searched on the web by age group

			**Age group**						
	**<40 yrs (N = 211)**		**40-54 yrs (N = 264)**		**>55 yrs (N = 42)**		**Total (N = 571)**		**p**
	**N**	**%**	**N**	**%**	**N**	**%**	**N**	**%**	
Disease characteristics	198	99.5	233	98.7	31	93.9	462	98.7	0.188
Diagnosis	178	92.7	191	86.4	25	86.2	394	89.1	0.061
Therapy	179	94.2	205	93.2	25	89.3	409	93.4	0.806
Nutrition	106	64	110	60.4	13	59.1	229	62.2	0.687
Physical activity	101	63.1	122	67.8	14	63.6	237	65.5	0.698
Specialty physicians or hospitals	175	91.6	190	88.8	24	82.8	389	89.6	0.481
Prevention of complications	155	88.1	173	86.9	17	70.8	345	86.5	0.026
Vaccines	87	56.1	77	46.4	5	29.4	169	50.0	0.073
Alterative therapy	118	70.7	100	57.1	12	54.5	230	63.2	0.018
Second opinion	116	71.2	123	66.8	11	55.0	250	68	0.179
Further pregnancy	108	64.7	71	43.6	9	50.0	188	54.0	0.000

The impact of online information is illustrated in Table 4. Respondents generally perceived that the information found on the Internet was useful and improved their understanding and management of the disease. Nearly 62% of parents also stated that information found online was useful for diagnosing their child’s disease. On the other hand, half of the parents stated that information found on the web increased their anxiety, and 11% that the information found made them change their physician. Twenty-four percent of respondents changed their child’s food habits as a result of information found in the web. Nearly 70% of respondents discussed the information found on the web with their physician.

**Table 4:** Impact of information search by age group

	**Age group**								
	**<40 yrs (N = 211)**		**40-54 yrs (N = 264)**		**>55 yrs (N = 42)**		**Total (N = 571)**		**p**
	**N**	**%**	**N**	**%**	**N**	**%**	**N**	**%**	
Increased my comprehension of the disease	170	86.7	180	78.3	24	75.0	374	81.7	0.048
Increased my anxiety	122	61.6	114	47.9	8	24.2	244	52.0	0.000
Improved the management of the disease	140	73.3	135	61.1	14	48.3	289	65.5	0.004
Made me change my son’s food habits	43	24.0	50	23.6	8	26.7	101	24.0	0.934
Was useful fo recognizing the diagnosis	138	69.3	138	57.5	15	45.4	291	61.7	0.006
Discussed with physicians the information I found	154	77.0	159	66.5	12	37.5	325	69.0	0.000
Changed physician	22	12.4	21	10.0	4	13.8	47	11.3	0.693

With respect to the impact of information found on the web on health decisions, we explored other factors associated with responders’ behavior through multivariate models. The results are illustrated in Table 5.

**Table 5:** Potential factors associated with health behavior

	**Useful to recognize the diagnosis**		**Increased my anxiety**		**Discussed with physician**		**Improved my comprehension of the disease**		**Improved the** **management of the disease**		**Changed food habits**	
	**OR 95% CI**	**p**	**OR 95% CI**	**p**	**OR 95% CI**	**p**	**OR 95% CI**	**p**	**OR 95% CI**	**p**	**OR 95% CI**	**p**
Age group (ref > =55 years)	-	-	-	-	-	-	-	-	-	-	-	-
40 - 54 years	1.58	0.297	1.22	0.713	2.38	0.072	0.75	0.598	2.00	0.182	-	-
	0.67 - 3.75		0.40 - 3.71		0.92 - 6.10		0.25 - 2.20		0.71 - 5.60			
< 40 years	2.32	0.108	1.40	0.604	3.11	0.048	1.05	0.941	3.77	0.028	-	-
	0.83 - 6.50		0.38 - 5.16		1.00 - 9.59		0.27 - 4.01		1.15 - 12.31			
Child age	1.00	0.916	0.96	0.043	0.99	0.435	0.98	0.269	1.02	0.236	-	-
	0.97 - 1.03		0.91 - 0.99		0.95 - 1.02		0.94 - 1.02		0.99 - 1.05			
Motor disability	-	-	1.38	0.092	-	-	-	-	-	-	1.30	0.384
			0.95 - 2.00								0.71 - 2.37	
Intellective disability	-	-	-	-	0.95	0.856	0.64	0.083	0.68	0.079	-	-
					0.58 - 1.58		0.39 - 1.06		0.44 - 1.05			
Social network account	-	-	0.70	0.090	2.80	0.046	-	-	-	-	-	-
			0.47 - 1.06		1.02 - 7.70							
Participate in forums	1.68	0.023	0.70	0.090	1.62	0.086	1.44	0.150	1.77	0.017	-	-
	1.08 - 2.63		0.47 - 1.06		0.93 - 2.83		0.87 - 2.39		1.11 - 2.81			
Communicates electronically with physician	-	-	-	-	1.93	0.004	1.39	0.236	1.37	0.165	1.13	0.685
					1.23 - 3.00		0.80 - 2.43		0.87 - 2.16		0.62 - 2.07	
Searched for nutritional info	-	-	-	-	1.12	0.693	-	-	-	-	3.50	0.000
					0.63 - 1.99						1.96 - 6.24	
Searched for future pregnancies	1.41	0.138	1.44	0.119	1.79	0.061	-	-	1.58	0.118	1.42	0.240
	0.89 - 2.24		0.91 - 2.28		0.97 - 3.30				0.88 - 2.84		0.78 - 2.61	
Searched for alternative therapies	1.81	0.054	1.20	0.402	2.26	0.040	2.06	0.08	1.38	0.236	1.83	0.097
	0.99 - 3.34		0.77 - 1.87		1.04 - 4.87		1.21 - 3.51		0.99 - 1.05		0.89 - 3.78	

Information found on the Internet was perceived more useful for recognizing their child’s disease by parents that participated in online forums. On the other hand, anxiety as a result of information found online was more frequent in parents of younger children. The likelihood of discussing information found on the Internet with their physician was higher in younger parents, in those who have a social network account, in those who communicate with their physician electronically, and in those who searched for alternative therapies. Parents who searched for alternative therapies more frequently stated that information found on the Internet increased their comprehension of their child’s disease. Parents who stated that online information improved their management of their child’s disease frequently participated in online forum and were younger. Finally, those who searched for nutritional information online frequently changed their child’s food habits.

## Discussion

Our study shows that Italian parents of patients with rare diseases are active Internet users, strongly engaged in information search and in online communities. These results have straightforward implications for the use of the Internet in their health care support.

Younger parents showed a more active profile on the Internet, although differences by age were not substantial. In particular, we did not observe any difference of note in the Internet behavior between parents younger than 40 years and those belonging to the 40–54 year age group. Younger parents frequently felt that information found on the web improved their management of their child’s disease and were more likely to discuss it with their physician. Respondents older than 55 years were less familiar with smart phones and Twitter, but their use of emails and Facebook was frequent. Social networks like Facebook are popular among people affected with chronic diseases (e.g. breast cancer or diabetes), and they can be a powerful tool for information dissemination, social support, awareness-raising, sharing of research issues, and fundraising [17,18].

Interestingly, a relatively high proportion of parents run a blog, in which they often report information about their child’s disease (data not shown).

Parents frequently participate in forums and online communities, where they mostly share information about their child’s disease. In the free comments accompanying the questionnaires, the positive role of communities online for self supporting was often emphasized (data not shown). Moreover, parents participating in online forums seem to perceive to better manage their child’s disease thanks to the information found on the Internet.

Our results suggest that parents of patients with rare diseases are avid information seekers on the Internet.

Gundersen showed that acquiring knowledge on their child’s disease is essential for parents to understand and adjust to an initially distressful condition [19]. More than half respondents search for alternative therapies on the web. These parents are often expert and curious Internet users, empowered by information found on the web, which they often discuss with their physicians.

Parents also frequently search for information on nutrition. Since parents searching for this information, according to our results, frequently change food habits, it is important that they discuss with their physician the adoption of appropriate nutritional habits.

A significant proportion of respondents also stated that they searched for information on diagnosis and that often the Internet was useful to recognize their child’s disease. Adults frequently go online specifically to figure out what medical condition they or someone else might have [5] and examples of self diagnoses of complex syndromes made by patients have been described [4].

Physicians should be ready to accept support and information from parents who have gathered details useful for diagnosis.

Of note, a large proportion of respondents searched online information for future potential pregnancies. Parents of patients with rare diseases may be at risk of genetic diseases or other problems that require accurate evaluation and a tailored preconception counseling [20]. We recently conducted a study on the validity and reliability of web information on preconception recommendations in which we found that valid and complete information is rarely found on the web [21]. Preconception counseling should be actively offered to all parents of patients with rare diseases by their physician.

The attitude of parents of patients with rare diseases to use the Internet for communicating with their physician is supported by the frequent use of email for exchanging information, and, less frequently, by the use of social networks for communication. Social networks can be an interesting opportunity for doctors for a timely and effective communication with parents of children with rare disease. Nevertheless, as stated by the American Medical Academy, doctors should be cautious in the use of social media for communication with patients and their families [22].

Of note, almost one half of respondents had a Skype account. Internet video telephony can be a powerful tool for communication with health professionals [23], telemonitoring, and also as a facilitating medium for hearing-impaired individuals [24]. Given its impact on a potential reduction of health care utilization and costs, video telephony may have a role in the communication between patients affected with rare diseases and health facilities.

Health information seeking and sharing on the Internet entails a high risk of incurring in imprecise information that may lead to unnecessary or harmful health behavior [25,26].

Since half respondents reported an increase in their anxiety as a result of online information seeking on their child’s disease, health professionals have a crucial role in its verification, validation and interpretation. In-person visits should include discussion on Internet use for health purposes, and trustable, certified websites should be systematically suggested to parents of children with rare diseases. Moreover, an active role of health professionals in online communities is still lacking. Active participation of physicians and other experts in online communities would result in an enhancement of validated information to families.

Our study is one of the few available on Internet use by parents of children with rare diseases. A recent work from Australia underlined the need of offering user-friendly online resources and of exploring information seeking and provision behaviors in this population [27]. Studies similar to ours may be useful to guide counseling of parents of children with rare diseases.

Our work has some limitations. The total number of emails sent by the UNIAMO associations to their members was estimated on the basis of the feedback we received by the associations. Therefore, we do not have a precise denominator for the response rate, which could be biased. Moreover, respondents participated in the survey on a voluntary base. Therefore, we likely selected parents with a higher interest in Internet use for health purposes. It might be that the attitude of parents of children with rare diseases to use the Internet for health purposes is lower than that observed in this study. Despite this bias, we can assume that Italian parents of patients with rare diseases use the Internet for health purposes more frequently compared to the general population.

## Conclusion

In conclusion, our findings show that information provided by the health care system through traditional channels is not sufficient to satisfy the knowledge needs of parents of children affected with rare diseases. Physicians, health care facilities and health agencies should take advantage of online interactions for empowering families of patients affected with rare diseases.

### Consent

The scope of the survey was explained in a letter that was sent by email to every participant. Informed consent to participate and use data for scientific purposes was obtained through the survey web form.

## Competing interests

The authors declare no financial or non-financial competing interest.

## Authors’ contributions

AET, RT and BD contributed to the conception, design and organization of the study and to the review and critique of the manuscript; FG, EA and MG contributed to data interpretation and drafted the manuscript; EP and EC contributed to the acquisition of data and performed the statistical analysis. All authors reviewed and accepted the final version of the manuscript.
